# IgG4-Related Pancreatitis and Immune Thrombocytopenia: A Case Report and Literature Review

**DOI:** 10.7759/cureus.1724

**Published:** 2017-09-28

**Authors:** Claire Sakiyama, Stephen Sullivan

**Affiliations:** 1 Faculty of Medicine, University of British Columbia, British Columbia, Canada; 2 Medicine/island Medical Program, University of Victoria, British Columbia, Canada

**Keywords:** autoimmune pancreatitis, immune thrombocytopenia, igg4 related disease

## Abstract

A patient with a prior diagnosis of IgG4-related autoimmune pancreatitis (AIP) presented four years later with severe prednisone resistant immune thrombocytopenia (ITP). Her case is reported and the scant literature of the very unusual possible association of IgG4-related AIP and ITP is reviewed. It is suggested that investigation for IgG4-related disease be part of the work-up of ITP.

## Introduction

IgG4-related disease (IgG4-RD) is a rare and recently recognized immune-mediated disorder characterized by tissue infiltration by IgG4-positive plasma cells. Most patients also have elevated serum IgG4. The commonest clinical manifestations are lymphadenopathy, submandibular gland enlargement, and autoimmune pancreatitis (AIP) but IgG4-RD is a systemic disease and can affect almost any organ [1-3]. We report a Japanese woman with a history of IgG4-related AIP who later developed severe immune thrombocytopenic purpura (ITP) resistant to treatment with prednisone. There are only a few reports of AIP and ITP occurring together, the literature of which we review.

## Case presentation

A 76-year-old Japanese woman presented with spontaneous bruising and a headache. Four years previously she was diagnosed with IgG4-related autoimmune pancreatitis (AIP) following a Whipple procedure for a presumed malignancy of the common bile duct. Histopathology was negative for malignancy, instead showing IgG4 autoimmune disease with large numbers of IgG4 secreting plasma cells in the pancreas, common bile duct, and gallbladder. She did not receive specific treatment for IgG4-RD following surgery and had no indication of disease progression. 

She had a several year history of dry eyes and enlarged submandibular salivary glands which had been biopsied and attributed to Sjögren’s syndrome. Her medical history included mild cognitive impairment secondary to a traumatic brain injury in her distant past and hypertension for which she was taking felodipine 5 mg/day. Four weeks before her presentation with bruising she had started pantoprazole 40 mg/day for postprandial epigastric pain.

On presentation with bruising and headache, she denied mucocutaneous, gastrointestinal, or genitourinary bleeding and her medications did not include antiplatelet or anticoagulant drugs. Her headache had persisted for over a day and was not associated with a history of trauma or signs or symptoms of increased intracranial pressure. She denied constitutional symptoms or abdominal pain. On examination, she had diffuse ecchymoses on her abdomen and all four limbs as well as petechiae on the lower limbs and small purpuric lesions in the oropharynx. The submandibular salivary glands were firm and enlarged. There was no lymphadenopathy. Neurological and abdominal examinations were normal.

Her platelet count was 3 x 109/L (normal = 150-450), hemoglobin 77 gm/L (normal = 120-160) and WBC of 5.2 x 109/L (normal = 4.5-11). Serum iron and transferrin saturation were low, 4 umol/L (normal = 10-30) and 8% (normal = 14-50) respectively. Ferritin was low-normal 33 ug/L (normal 20-160) and vitamin B12 was normal. INR and PTT were 1 (normal ≤1.1) and 24 sec (normal = 25-35), respectively. Liver biochemistry revealed total bilirubin 25 umol/L (normal = 8-22), alkaline phosphatase 140 U/L (normal = 35-100) and ALT 17 U/L (normal = 10-55). Total gammaglobulin was 26.1 g/L (normal = 6-16). IgG subclasses were measured. IgG4 was very high at 15.4 g/L (normal = 0.05-1.25). IgG1 was 15.5 g/L (normal = 2.8-8.0), IgG2 was 16.2 g/L (normal = 1.15-5.7) and IgG3 was normal at 0.85 g/L (normal = 0.24-1.25). The pathologist noted this to be strongly suggestive of IgG4-RD. Serology for Helicobacter pylori, hepatitis B and C, and HIV was negative. 

**Figure 1:**
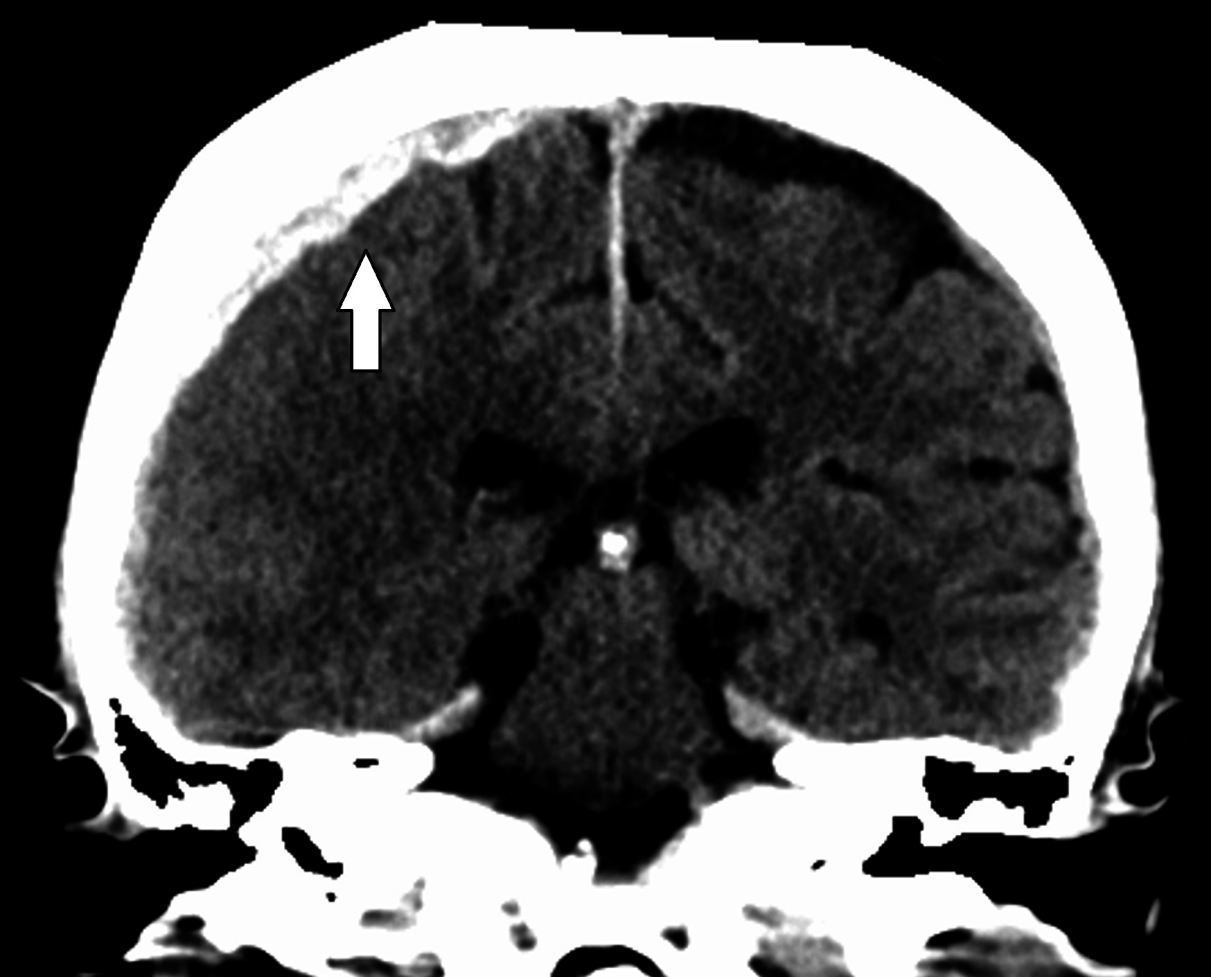
Subdural hematoma

A non-contrast computed tomography (CT) scan of the head showed an acute right subdural hematoma 0.7 cm in maximum thickness and a small, subacute, left subdural hematoma measuring 2-3 mm (Figure 1). CT of the abdomen did not identify any abnormalities of the pancreas or biliary system other than air in the biliary tree consistent with the patient’s patent enterostomy.

The patient was initially thought to have pantoprazole induced ITP [4] complicated by a spontaneous intracranial hemorrhage. She was admitted. The pantoprazole was stopped. She received intravenous immunoglobulin (IVIG) 1 gm/kg, one unit of platelets, and prednisone 50 mg per day, orally. On the fourth day her platelets were 66 x 109/L, but on the fifth day they began to decline, returning to 3 x 109/L by the tenth day. Bone marrow biopsy was negative for dysplasia and malignancy, and showed adequate megakaryocytes. On the fourteenth day of admission her platelets were still only 5 x 109/L. She declined splenectomy and was therefore started on azathioprine 50 mg per day orally. A prednisone taper was started at that time. Within six days her platelets had risen to 18 x109/L. On her 22nd day of admission, her platelets were 36 x 109/L and she was discharged with outpatient follow-up. Nineteen months after presentation her platelet count was 91 x 109/L on prednisone 5 mg and azathioprine 100 mg daily.

## Discussion

IgG4-RD is a pleomorphic disease with involvement described in over 40 tissues and organs [1-3]. Autoimmune pancreatitis is one of its commonest presentations. Is ITP part of the picture? We believe it is too early to tell. Including ours, there are only ten reports of AIP and ITP occurring in the same patient [5-13] (Table 1). In the seven cases where it was measured, the AIP was recognized to be IgG4 related. Several patients had or later developed other autoimmune phenomena including Sjögren's syndrome [8], primary biliary cirrhosis [13], hypophysitis [14], lung involvement [8, 10, 14], periaortitis [12], and renal disease [7, 15]. In several cases, like in ours, the development of ITP was delayed, occurring months to years after the diagnosis of AIP. All patients were Japanese and all but one were men. Ohara, et al. [16] mention two cases of AIP and ITP apparently reported in Western patients and reference a paper by Zamboni, et al. [17]. However, a careful reading of that paper and personal contact with its two senior authors failed to confirm those cases. None of the previous cases reported ITP resistant to treatment with prednisone, although one required the addition of Helicobacter (H.) pylori eradication to achieve remission of the ITP [13], and one was treated solely with H. pylori eradication [11]. However, we did find a report of a 40-year-old Moroccan patient with IgG4-related kidney disease, but without evidence of pancreatitis, who four years later developed ITP resistant to steroids, IVIG, and cyclophosphamide, requiring a splenectomy [18].

**Table 1:** Cases of AIP complicated by ITP AIP = autoimmune pancreatitis; ITP = immune thrombocytopenia; *Hp* = *Helicobacter pylori*; PBC = primary biliary cirrhosis; MPGN = membranoproliferative glomerulonephritis; TIN = tubulointerstitial nephritis; IVIG = intravenous immunoglobulin; NR = not reported.

Author [ref]	Age/sex	Time from AIP to ITP	IgG4 mg/dl	Therapy for ITP	Other organ involvement
Seko [5]	66/M	14 days	NR	Steroids	Uveitis age 69, lymphocytic hypophysitis and organizing pneumonia at 75 [14]
Nakamura [6]	78/M	6 months	900	Steroids	
Akashi [7]	61/M	6 years	NR	Steroids	Interstitial nephritis
Nakazawa [8]	70/M	1 month	NR	Steroids	Pulmonary fibrosis, biliary hilar stricture, positive Schirmer's test
Fukushima [9]	80/M	10 days	553	Steroids	MPGN and TIN 14 months later [15]
Murase [10]	73/M	same time	468	Steroids	Interstitial pneumonia
Miyatani [11]	64/M	4 months	337	eradication	
Sawai [12]	67/M	28 months	462	Steroids	Periaortitis
Takasumi [13]	63/F	same time	160	Steroids and eradication	PBC, submandibular gland enlargement
Our case	76/F	4 years	1540	Steroids, IVIG, azathioprine	Sjögren's syndrome

If ITP is part of the spectrum of IgG4-RD, what is the role of IgG4 in its genesis? IgG4 is not the usual subclass of IgG associated with ITP [19]. Only one paper has addressed the question finding that the antiplatelet antibody appeared to be IgG4 [10]. Clearly, there is more work to be done.

## Conclusions

Our case adds to the scant literature on ITP occurring with AIP and with IgG4-RD. It is the second patient reported thus far in whom the ITP was resistant to first-line treatment with prednisone and IVIG; although it is well recognized that not all cases of ITP respond to standard therapy, nor are they all "idiopathic". Perhaps the measurement of serum IgG4 should be part of the "routine" investigation of ITP just as checking for *H. pylori* has become.

Finally, our report and others remind us that those caring for patients with an IgG4-related disease should be vigilant for the development of synchronous and metachronous complications in other organ systems. 
